# The Role of MCPIP1 in Macrophage Polarization and Cardiac Function Post‐Myocardial Infarction

**DOI:** 10.1002/advs.202500747

**Published:** 2025-04-26

**Authors:** Xingxu Zhang, Yuan Fang, Xiaoming Qin, Yiwei Zhang, Bo Kang, Li Zhong, Baoxin Liu, Jiachen Luo, Yidong Wei

**Affiliations:** ^1^ Department of Cardiology Shanghai Tenth People's Hospital Tongji University School of Medicine 301 Middle Yanchang Road Shanghai 200072 China; ^2^ Department of Health Policy and Management Fielding School of Public Health University of California 650 Charles E Young Dr S Los Angeles CA 90095 United States; ^3^ College of Osteopathic Medicine of the Pacific Western University of Health Sciences 309 E. Second Street Pomona CA 91766 United States

**Keywords:** ferroptosis, Heart‐spleen axis, macrophage, MCPIP1

## Abstract

Macrophages play a critical role in both initiating and resolving inflammation following MI (myocardial infarction). Their polarization is essential for maintaining cardiac function. This study aims to explore the role of MCPIP1(Monocyte chemotactic protein‐induced protein 1) in regulating macrophage polarization and its impact on heart‐spleen interactions during MI recovery. The role of MCPIP1 was investigated using histological staining, RNA sequencing of bone marrow‐derived macrophages, co‐culture experiments, and validated by western blot. Compared to controls, myeloid MCPIP1‐deficient mice had lower survival rates, larger infarction areas, and more severe inflammatory responses after MI. This was due to increased M1 polarization and impaired conversion to the M2 phenotype. Ferroptosis activation in MCPIP1‐deficient macrophages was inhibited by Fer‐1 and PFT‐α, which promoted M2 polarization and fibroblast activation into myofibroblasts. MCPIP1‐deficient MI mice also showed splenomegaly and elevated levels of circulating macrophages, indicating excessive extramedullary hematopoiesis. Splenectomy improved survival rates and reduced infarction size in MCPIP1‐deficient mice. MCPIP1 suppresses the P53/ferroptosis pathway to regulate macrophage polarization and TGF‐β/SMAD3‐mediated fibroblast activation. Its deficiency exacerbates inflammation through abnormal splenic macrophage output, impairing cardiac repair. MCPIP1 is a promising therapeutic target for modulating ferroptosis and heart‐spleen communication to protect cardiac function following MI.

## Graphical abstract

Following myocardial infarction, MCPIP1 deficiency in macrophages triggers ferroptosis, driving proinflammatory M1 dominance over reparative M2 phenotypes. This imbalance amplifies splenic monocyte release and sustains cardiac inflammation. Concurrently, excessive inflammatory mediators and insufficient TGF‐β impair fibroblast activation, delaying extracellular matrix repair. These cascades culminate in cardiac dysfunction, elevated heart rupture rates, and reduced survival in MCPIP1‐ deficient mice.

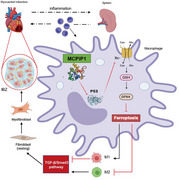

## 1 Introduction

Myocardial infarction (MI) remains a leading cause of death and disability worldwide, imposing a substantial health and economic burden on society.^[^ ^1^ ^]^ Following MI, a complex inflammatory response is initiated, involving a diverse array of various immune cells and inflammatory factors.^[^ ^2^, ^3^ ^]^ Obtaining an equilibrium between pro‐inflammatory and anti‐inflammatory processes can profoundly affects cellular functions within the inflamed myocardial tissue.^[^ ^4^ ^]^ Proper resolution of inflammation is essential for a favorable healing outcome, as excessive or prolonged inflammatory response can lead to adverse remodeling and complications.^[^ ^5^ ^]^ Consequently, maintaining a delicate balance between pro‐inflammatory and anti‐inflammatory processes is crucial for removing damaged cardiac tissue and preserving heart function.^[^ ^6^, ^7^ ^]^

In MI, the timely recruitment and multifaceted functions of monocytes and macrophages drive both the inflammatory response and subsequent tissue repair. Monocytes and macrophages are recruited to the infarcted area in response to damage‐associated molecular patterns (DAMPs) and inflammatory cues released by dying cardiac cells.^[^ ^8^ ^]^ These cells assist in removing necrotic debris and secrete cytokines that shape the inflammatory milieu. They also help form tension‐free zones in the ischemic and necrotic myocardium and activate fibroblasts, converting them into myofibroblasts that stabilize the extracellular matrix (ECM) through collagen deposition.^[^ ^9^, ^10^ ^]^ This adaptive remodeling prevents heart rupture during the acute phase of MI.^[^ ^11^ ^]^ During this process, macrophages play as dual role: acting as “arsonists” by releasing pro‐inflammatory factors that drive inflammation and as “firefighters” by producing repair factors that promote tissue healing.^[^ ^8^ ^]^ Given their high heterogeneity and plasticity, the regulation of macrophages function has become an attractive therapeutic strategy for improving long‐term prognosis in MI patients.^[^ ^12^, ^13^, ^14^, ^15^ ^]^

Monocyte chemoattractant protein‐induced protein 1 (MCPIP1), a member of CCCH zinc finger family, is increasingly recognized as a pivotal regulator of inflammation.^[^ ^16^ ^]^ This protein was first identified in peripheral blood monocytes from patients with ischemic heart disease in 2006.^[^ ^17^ ^]^ MCPIP1 expression increases in response to LPS/IL‐1β activation of the NFκB pathway via TLR receptors.^[^ ^18^ ^]^ Through its ribonuclease activity, MCPIP1 then degrades mRNAs encoding several pro‐inflammatory cytokines (e.g., IL‐6, IL‐1β, IL‐2, IL‐12, and IL‐17), thereby exerting an anti‐inflammatory effect.^[^ ^19^ ^]^ Mice lacking MCPIP1 exhibit severe inflammation and premature death.^[^ ^20^ ^]^ Overexpression of MCPIP1 in cardiomyocytes confers protection against LPS‐induced myocarditis.^[^ ^21^ ^]^

Despite these findings, the roles of MCPIP1 in regulating post‐MI inflammation remains underexplored. To bridge this gap, we generated a myeloid‐specific MCPIP1 knockout mouse model to investigate the role of MCPIP1 in macrophage function during MI. Our findings provide insights into a potentially novel therapeutic strategy for modulating the inflammatory response after MI.

## 2 Results

### 2.1 MCPIP1 is Highly Expressed in Macrophages Following MI

**Figure 1:**
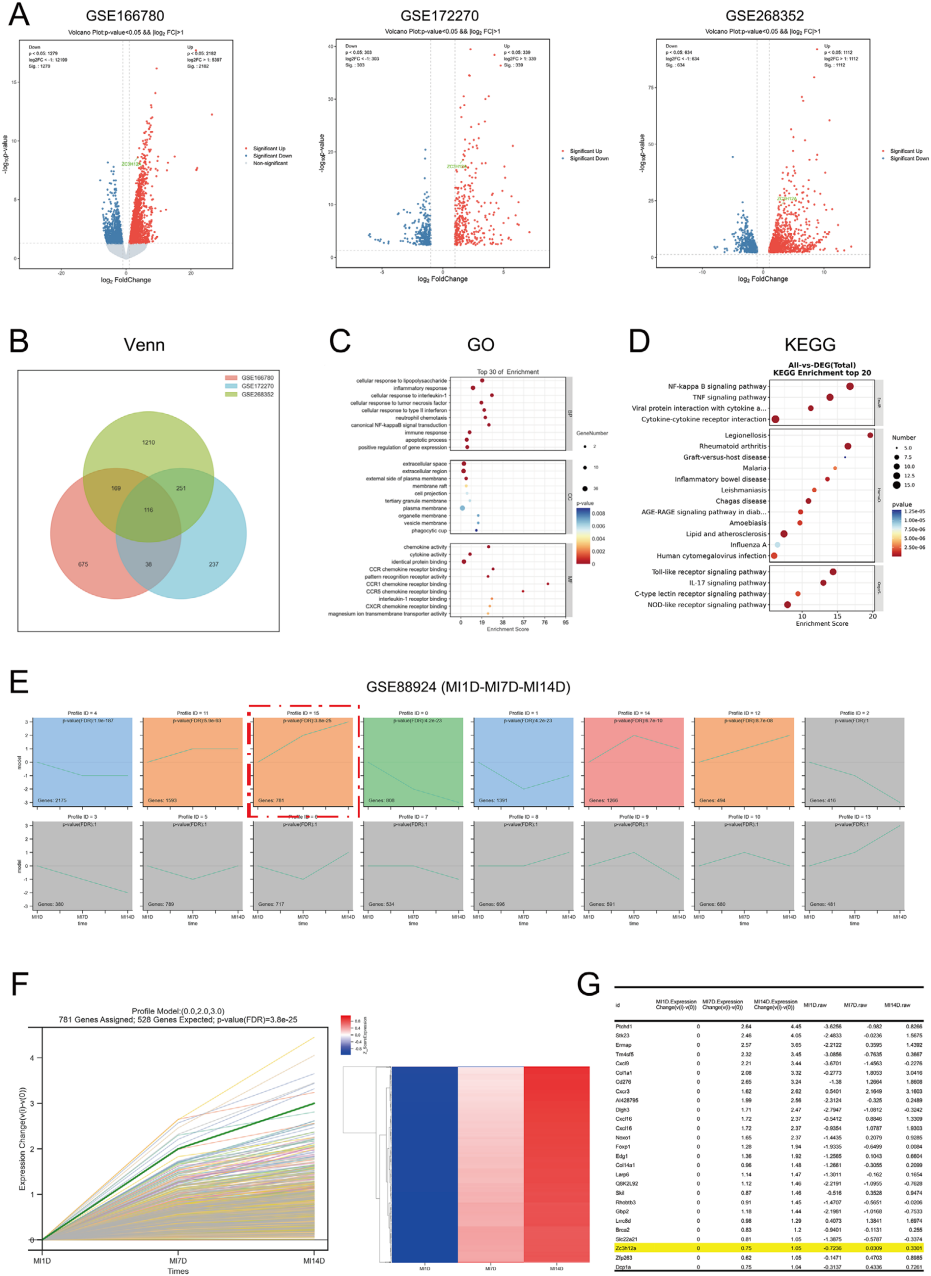
MCPIP1 Is Expressed In Macrophages After Myocardial Infarction and Its Expression Increases Over Time. A) Volcano plot of differential genes, utilizing three datasets from GEO: GSE166780 (comparing peripheral blood monocytes from AMI patients versus healthy individuals), GSE172270 (comparing monocyte‐derived macrophages from AMI patients versus healthy individuals), and GSE268352 (comparing LPS‐treated versus PBS‐treated human monocyte‐derived macrophages), with Zc3h12a (MCPIP1) highlighted in green. B) Venn contains 116 differential genes (DEGs). GO C) and KEGG D) analyses were conducted using DSEGs. E) Utilizing GSE88924 from GEO, a STEM (Short Time‐series Expression Miner) trend analysis was performed on the gene expression profiles of macrophages in the mouse heart, following the temporal sequence of MI1D, MI7D, and MI14D. F) Module 15, which exhibits significant trend differences, contains 528 genes. The green line represents the expression trend of MCPIP1 over time. G) This figure displays a subset of genes within Module 15, with Zc3h12a (MCPIP1) highlighted in yellow.

To explore the gene expression profile within macrophages after MI, we analyzed three representative GEO datasets. We found that MCPIP1 was upregulated in all three datasets (**Figure** **1**A). A total of 116 differentially expressed genes overlapped among these datasets (Figure 1B). GO and KEGG analyses revealed strong association with inflammatory responses and classical inflammatory pathways (Figure 1C,D). STEM analysis demonstrated progressively increase in MCPIP1 expression as MI progressed (MI 1D – 7D – 14D). Such increase is particularly with third module, by exhibiting significant temporal expression differences (Figure 1E–G).

**Figure 2:**
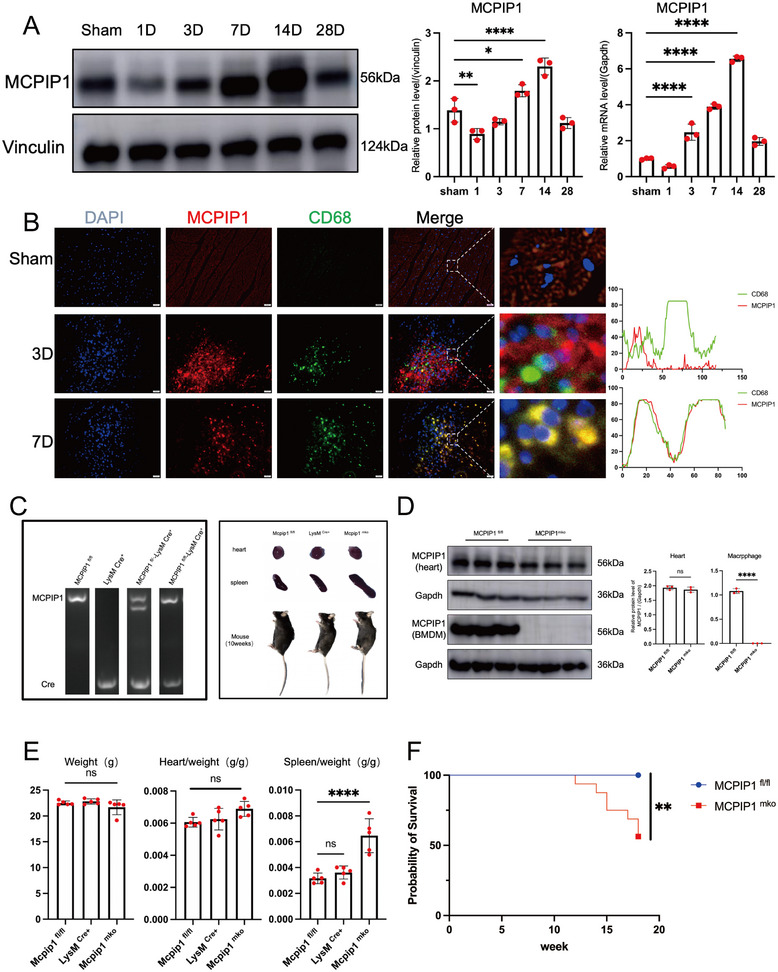
Post‐Myocardial Infarction, MCPIP1 is Primarily Expressed by Macrophages. A) MCPIP1 protein and mRNA levels at various time points (sham, 1, 3, 7, 14, and 28 days) post‐MI. *n* = 3. B) IF of heart at various time points (sham, MI3D, and MI7D), DAPI (blue) stains nuclei, MCPIP1 (red), and CD68 (green) stains macrophages. The line graph represents the distribution of red (MCPIP1) and green (CD68) fluorescence within the tissue sections.(C) Genotype verification of myeloid‐specific MCPIP1 knockout mice. D) Protein levels of MCPIP1 in heart and BMDMs from MCPIP1 ^mko^ and MCPIP1 ^fl/fl^ mice. *n* = 3. E) Body weight, heart‐to‐body weight ratio, and spleen‐to‐body weight ratio in littermate mice of the same age (10 weeks). *n* = 5. F) Survival curves of unoperated MCPIP1^mko^ and MCPIP1^fl/fl^ mice. *n* = 15. ns *P* > 0.05, ^*^ *P* < 0.05, ^**^ *P* < 0.01, ^***^ *P* < 0.001, ^****^ *P* < 0.0001.

To validate the bioinformatics analysis, we induced MI in wild‐type (WT) mice. Compared to the sham group, MCPIP1 protein and mRNA levels have more increased progressively over time, peaking on Day 14 (**Figure** **2**A). Previous studies have shown that MCPIP1 is not exclusive to macrophages.^[^ ^22^, ^23^, ^24^ ^]^ Dual IF staining using CD68 (a specific marker for macrophages) and MCPIP1 confirmed that MCPIP1 was highly expressed by macrophages (CD68^+^ cells) in the infarct border zone (IBZ) on Day 7 post‐MI (Figure 2B), aligning with silico results (Figure 1E).

### 2.2 MCPIP1 ^mko^ Mice Exhibit Higher Mortality and Poorer Cardiac Function Following MI

Next, we generate MCPIP1 ^mko^ mice to explore the functional role of MCPIP1 in regulating macrophage inflammation after MI (Figure 2C). WB confirmed efficient deletion of MCPIP1 in heart tissue and bone marrow‐derived macrophages (BMDMs) (Figure 2D). During breeding, MCPIP1 ^mko^ mice progressively developed systemic inflammatory responses, leading to splenomegaly around 10 weeks of age (Figure 2E), and increased mortality by 12 weeks (Figure 2F).

**Figure 3:**
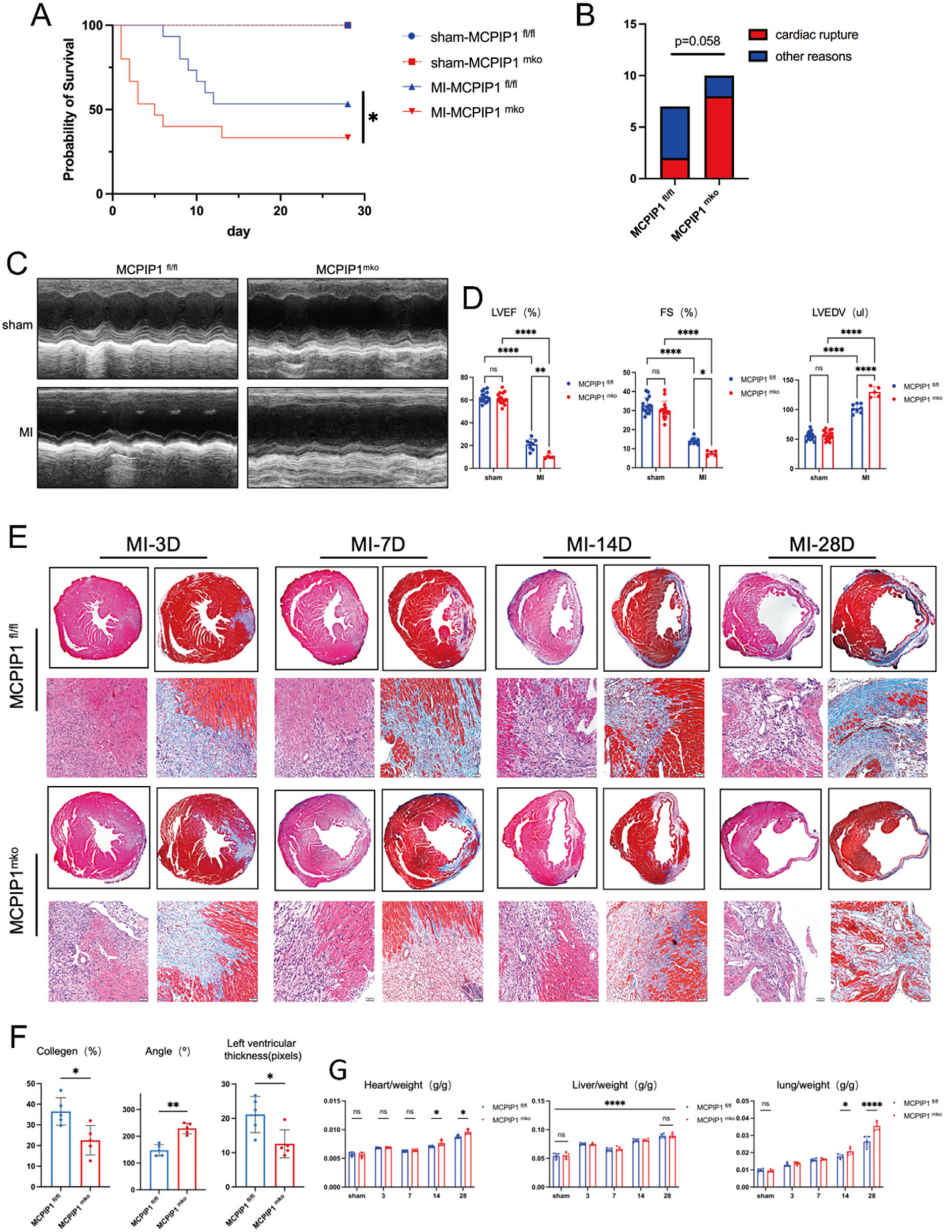
MCPIP1 ^mko^ Mice Exhibit Higher Mortality and Poorer Cardiac Function Following MI. A) Survival curves of MCPIP1^mko^ and MCPIP1^fl/fl^ mice after MI. *n* = 15. B) Causes of death are categorized as cardiac rupture and other causes. C) Representative cardiac ultrasound images at MI28D. D) Echocardiographic measurements of LVEF, FS, and LVEDV in mice. At least 5 mice per group. E) MASSON and HE staining of hearts from MCPIP1^mko^ and MCPIP1^fl/fl^ mice at different time points (3, 7, 14, and 28 days) post‐MI. F) Cardiac collagen content, infarction angle, and left ventricular thickness in MCPIP1^mko^ and MCPIP1^fl/fl^ mice at MI28D based on MASSON staining results. *n* = 5. G) Comparison of wet organ weights (heart, liver and lungs) relative to body weight at various time points. *n* = 5.ns *P *> 0.05, ^*^ *P* < 0.05, ^**^ *P* < 0.01, ^***^ *P* < 0.001, ^****^ *P* < 0.0001.

To avoid confounding effects from spontaneous inflammation in older MCPIP1 ^mko^ mice, MI was induced at 6 weeks of age. Over a 28‐Day period, the survival rate of MCPIP1 ^mko^ mice (33.33%) was significantly lower than controls (53.33%) (**Figure** **3**A). Necropsy revealed a higher incidence of cardiac rupture in MCPIP1 ^mko^ mice (8/10, 80.00%), although this difference did not reach statistically significant (P = 0.058) (Figure 3B). Echocardiography at 28 days post‐MI showed worse cardiac function in MCPIP1 ^mko^ mice, with significantly lower LVEF% and FS%, plus increase in LVEDV (Figure 3C,D). Histological examination at multiple time points (3, 7, 14, and 28 days post‐MI) demonstrated loosely arranged myocardial tissue, prominent vacuolar structures, and reduced collagen deposition in MCPIP1 ^mko^ mice (Figure 3E). The MCPIP ^mko^ mice also presented larger infarction angle, thinner left ventricles, and overall lower collagen content than their controls (Figure 3F). Previous studies have shown that MI can lead to heart failure and damage to target organs, including the heart, liver, and lungs.^[^ ^25^, ^26^, ^27^ ^]^ Compared to controls, MCPIP1 ^mko^ mice exhibited higher ratios heart, spleen, and lung weights to body weight, respectively (Figure 3G).

Collectively, these data suggest that MCPIP1 ^mko^ mice suffer from increased mortality and impaired cardiac remodeling following MI.

### 2.3 MCPIP1 Deficiency Leads to Excessive Macrophage Accumulation

**Figure 4:**
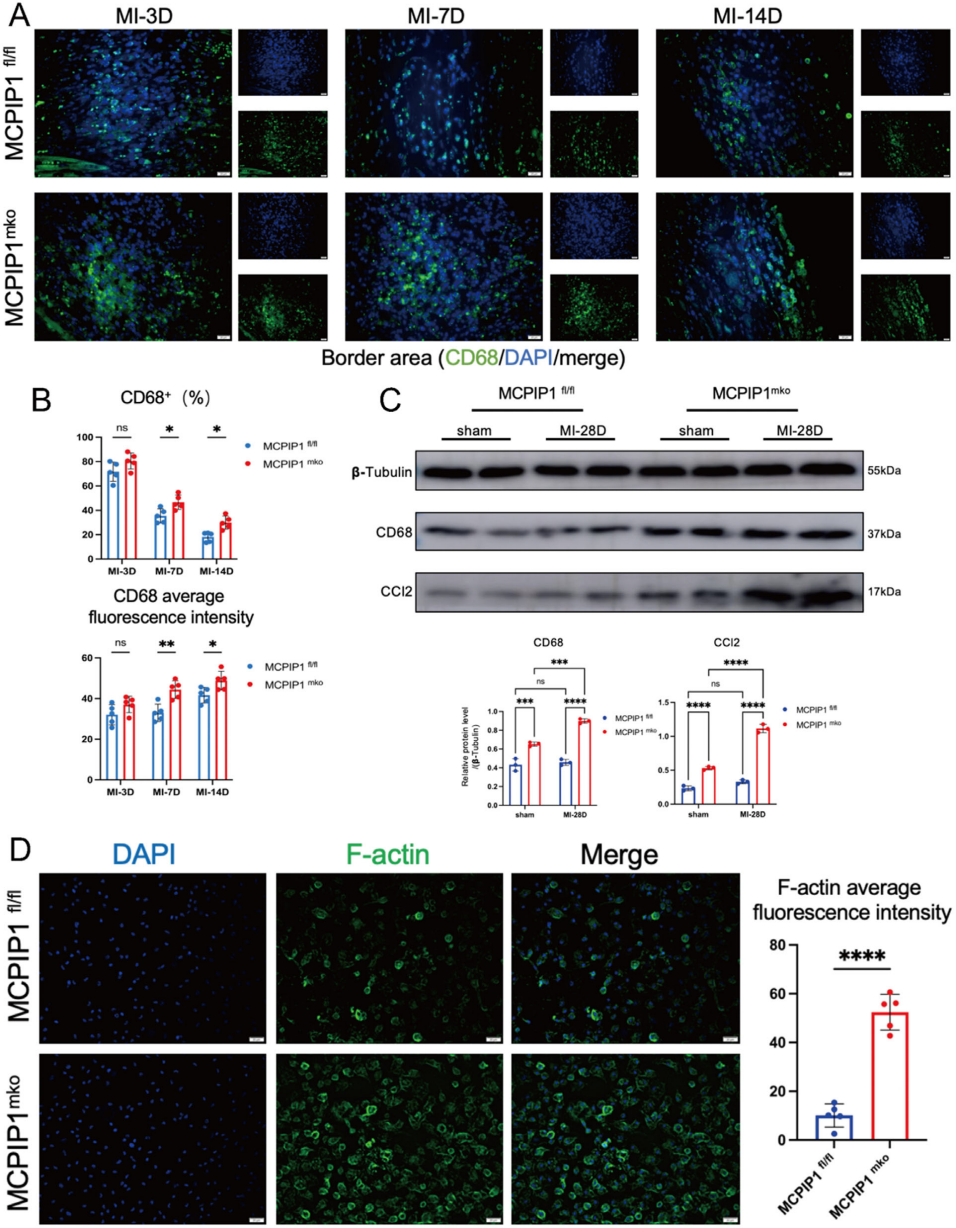
MCPIP1 Deficiency Leads to Excessive Macrophage Accumulation. A,B) IF showing the number of macrophages (CD68+) and mean fluorescence intensity in IBZ at different time points (3, 7, 14 days) post‐MI. DAPI (blue) stains nuclei, and CD68 (green) stains macrophages. *n* = 5 fields of view. C) Protein levels of CD68 and CCL2 in IBZ of mice hearts after MI. *n* = 3. D) Phalloidin staining revealed the content of F‐actin in BMDMs from MCPIP1^mko^ and MCPIP1^fl/fl^ mice. DAPI (blue) stains nuclei, and F‐actin (green) stains filamentous actin. *n* = 5 fields of view. ns *P* > 0.05, ^*^ *P* < 0.05, ^**^ *P* < 0.01, ^***^ *P* < 0.001, ^****^ *P* < 0.0001.

We are curious whether MCPIP1 deficiency macrophages will accumulate within the infarcted heart. The higher number of CD68^+^ cells and fluorescence intensity in MCPIP1 ^mko^ mice hearts confirm excessive macrophage infiltration into the IBZ (**Figure** **4**A,B). WB revealed increased levels of CD68 and CCL2(also known as MCP‐1) in MCPIP1 ^mko^ mice hearts, indicating elevated macrophage accumulation and enhanced chemotactic signals (Figure 4C). Furthermore, IF staining for F‐actin in BMDMs showed upregulated cytoskeletal proteins in MCPIP1‐deficient macrophages, suggesting greater migration capacity (Figure 4D). MCPIP1 profoundly alters the chemotactic ability of macrophages, causing them to continuously accumulate in the heart after infarction.

### 2.4 MCPIP1 ^mko^ Macrophages Remain Predominantly in M1 Polarization, Leading to Excessive Inflammation and Impaired Collagen Production

**Figure 5:**
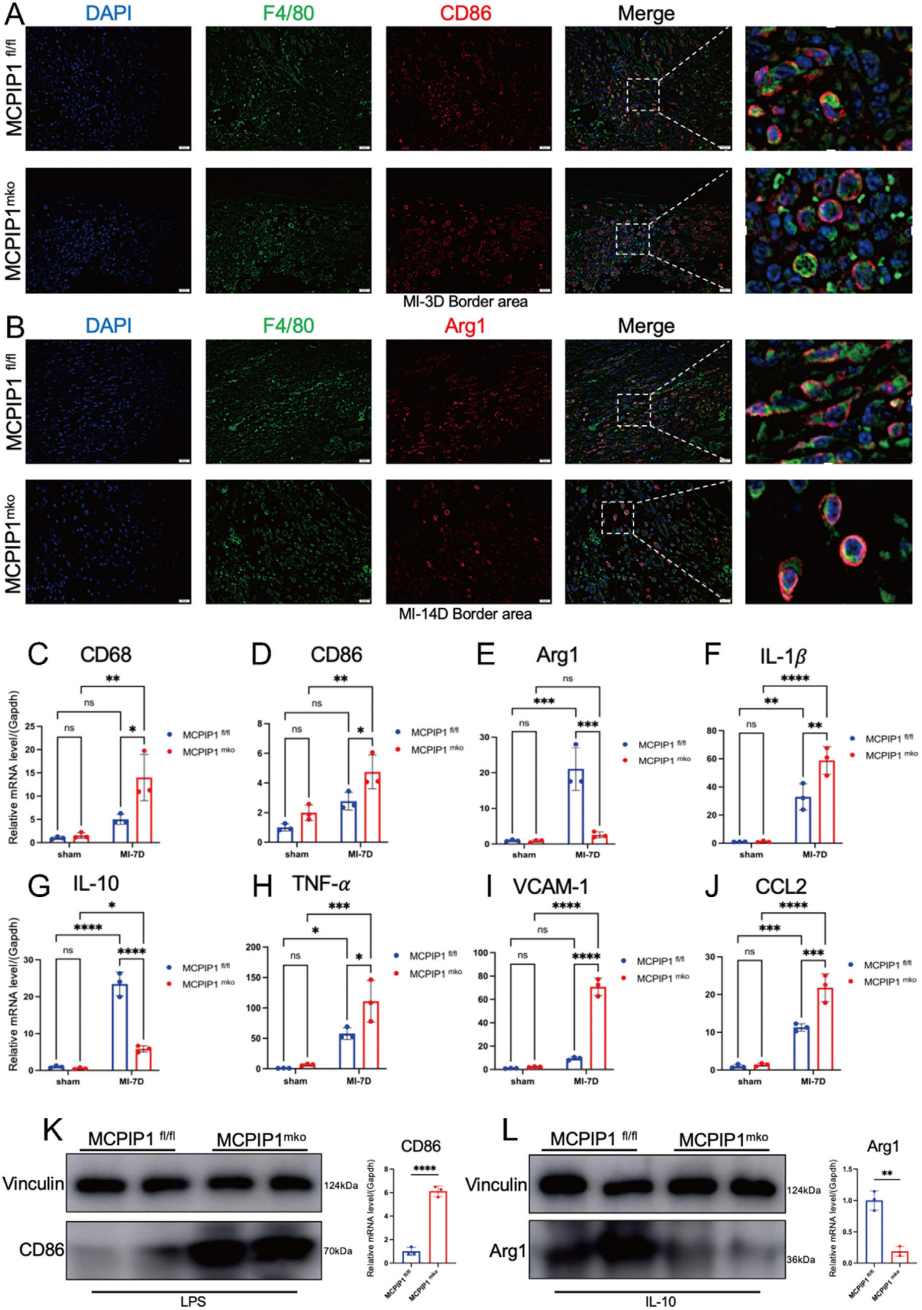
MCPIP1 Plays A Crucial Role in Regulating M2 Polarization Of Macrophages And Cardiac Inflammation. A) IF reveals the percentage of M1 macrophages in IBZ of the hearts. The percentage of (F4/80 + CD86+)/ F4/80+ was calculated. CD86 serves as an M1‐specific marker, while F4/80 is a macrophage‐specific marker. DAPI (blue), F4/80 (green), CD86 (red). *n* = 5 per field of view. B) IF shows the percentage of M2 macrophages in IBZ of the hearts. The percentage of (F4/80 + Arg1+)/ F4/80+ was calculated. Arg1 is an M2‐specific marker, and F4/80 is a macrophage‐specific marker. DAPI (blue), F4/80 (green), Arg1 (red). *n* = 5 per field of view. mRNA was extracted from the heart tissues of mice in the sham group and 7 days post‐MI. qRT‐PCR was performed to detect the relative mRNA levels of CD68C), CD86D), Arg1E), IL‐1βF), IL‐10G), TNF‐a H), VCAM‐1I) and CCL2 J). *n* = 3. BMDMs from MCPIP1^mko^and MCPIP1^fl/fl^ mice were treated with LPS (50 ng mL^−1^) and IL‐10 (20 ng mL^−1^) respectively to induce classical M1 and M2 phenotypes. WB and qRT‐PCR showed protein and mRNA of CD86 (K) and Arg1(L). *n* = 3. ns *P* > 0.05, ^*^ *P* < 0.05, ^**^ *P* < 0.01, ^***^ *P* < 0.001, ^****^ *P* < 0.0001.

Macrophages exhibit significant heterogeneity, with M1 macrophages (marked by CD86) promoting inflammation and M2 macrophages (marked by Arg1) facilitating tissue repair. IF staining at two time points post‐MI—the acute inflammatory phase (MI‐3 Days) and the scar repair phase (MI‐14 Days)—revealed that MCPIP1 ^mko^ mice had a higher proportion of M1 macrophages during the acute phase (p = 0.056) and a severe deficiency of M2 macrophages during the repair phase (**Figure** **5**A,B). Macrophage markers (CD68) (Figure 5C) and M1 phenotype markers (CD86) (Figure 5D) were elevated, while M2 phenotype markers (Arg1) (Figure 5E) were reduced in MCPIP1 ^mko^ mice hearts. Furthermore, qRT‐ PCR indicated upregulation of pro‐inflammatory markers (IL‐1β, TNF‐α) (Figure 5F,H) and chemokines (CCL2, VCAM‐1) (Figure 5I,J), while showed downregulation of repair‐associated genes (IL‐10) in MCPIP1 ^mko^ hearts (Figure 5G). In vitro, MCPIP1‐deficient macrophages exhibited exaggerated CD86 expression under LPS stimulation but failed to upregulate Arg1 in response to IL‐10(Figure 5K,L), confirming the crucial role of MCPIP1 for M2 polarization.

**Figure 6:**
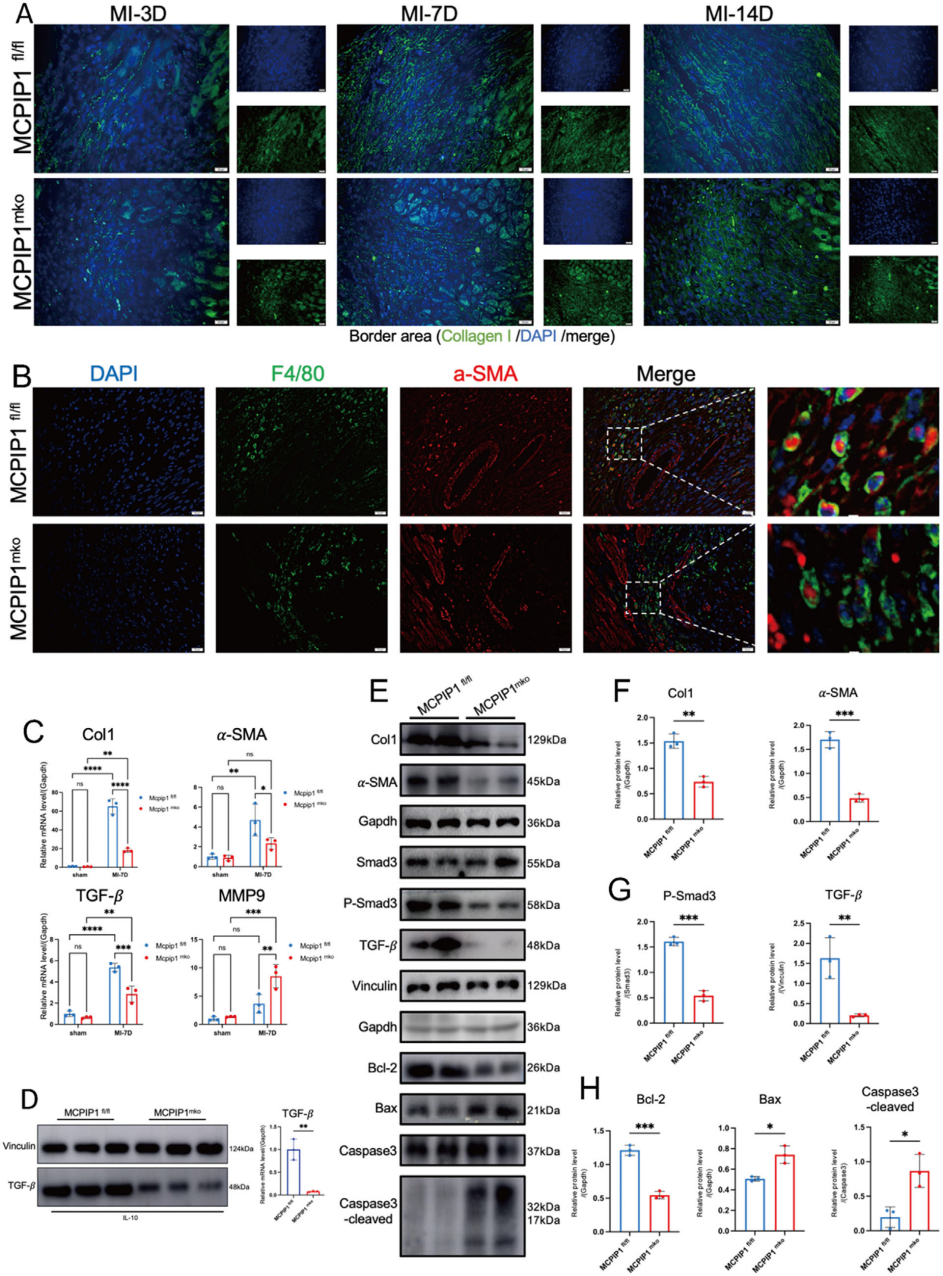
Deficiency of MCPIP1 in Macrophages Suppresses Cardiac Fibroblast Activation. A) IF displays the content of type I collagen in IBZ of MCPIP1^mko^ and MCPIP1^fl/fl^ hearts at different time points post‐MI (3, 7, and 14 days), DAPI (blue), Collagen I (green). *n* = 5 per field of view. B) IF demonstrates MMT in infarcted hearts, calculated as the percentage of (a‐SMA+ F4/80+)/ F4/80+. DAPI (blue), F4/80 (green), a‐SMA (red). *n* = 5 per field of view. C) qRT‐PCR was performed to detect the relative mRNA levels of Col1, α‐SMA, TGF‐β, and MMP9. *n* = 3. D) WB was performed to detect the protein level of TGF‐β in BMDMs from MCPIP1 ^mko^ and MCPIP1 ^fl/fl^ mice after IL‐10 treatment. *n* = 3. E) BMDMs from MCPIP1 ^mko^ and MCPIP1 ^fl/fl^ mice were co‐cultured with PCFs from WT mice, respectively. WB analysis was conducted to assess the protein levels of myofibroblast markers F), TGF‐β/Smad3 signaling pathway G) and apoptosis related proteins (H), *n* = 3. ns *P* > 0.05, ^*^ *P* < 0.05, ^**^ *P* < 0.01, ^***^ *P* < 0.001, ^****^ *P* < 0.0001.

Because of M2 macrophages produce TGF‐β to stimulate myofibroblast‐mediated collagen deposition,^[^ ^28^, ^29^ ^]^ MCPIP1 ^mko^ mice presented reduced collagen I secretion in the IBZ (**Figure** **6**A). Fibroblast activation markers (Col I, α‐SMA, TGF‐β) were decreased, and ECM‐degrading enzymes (MMP9) were increased in MCPIP1 ^mko^ mice hearts (Figure 6C). The TGF‐β/Smad3 signaling pathway is essential for fibroblast activation into myofibroblasts and subsequent collagen I secretion.^[^ ^29^ ^]^ MCPIP1‐deficient macrophages, even under IL‐10 stimulation, failed to secrete normal levels of TGF‐β (Figure 6D). WB analysis indicated that MCPIP1‐deficient macrophages did not effectively activate primary cardiac fibroblasts (PCFs) to myofibroblasts (Figure 6E,F) via the TGF‐β/Smad3 pathway (Figure 6E,G), resulting in decreased collagen production (Figure 6F) and increased PCFs apoptosis (Figure 6H). Furthermore, co‐staining with α‐SMA (myofibroblast marker) and F4/80 (macrophage marker) revealed reduced macrophage‐to‐myofibroblast transition (MMT) in the infarcted hearts of MCPIP1 ^mko^ mice (Figure 6B).

Collectively, these findings underscore that MCPIP1 deficiency exacerbates inflammation and impairs myocardial repair.

### 2.5 MCPIP1 Inhibits Ferroptosis by Restraining P53 in Macrophages

**Figure 7:**
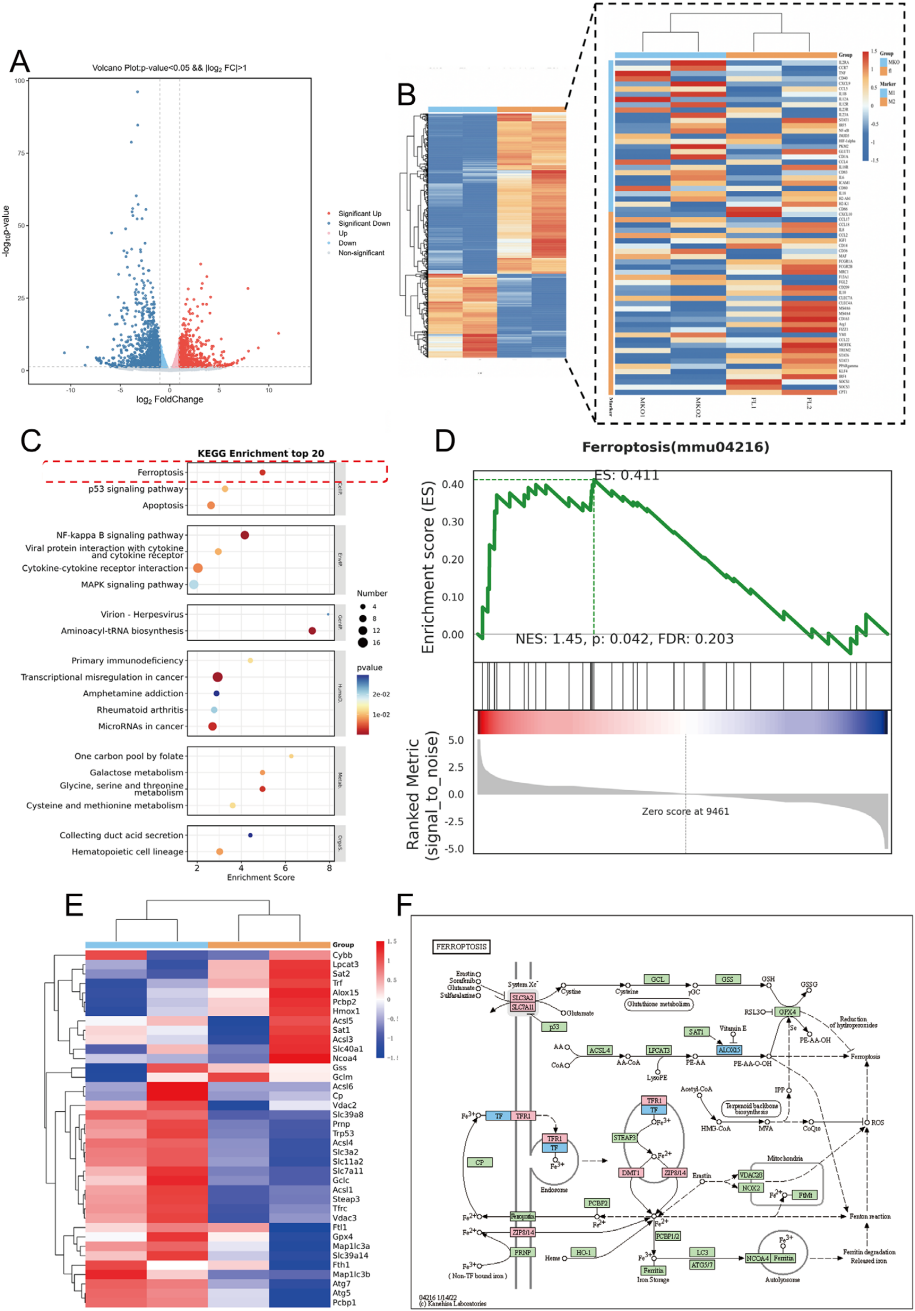
Ferroptosis in Macrophages Lacking MCPIP1 is Significantly Enhanced. RNA‐seq analysis was performed on BMDMs from MCPIP1 ^mko^ and MCPIP1 ^fl/fl^ mice, and volcano plots A), heatmaps B), and KEGG) enrichment pathway maps C) were generated based on the differential gene expression. GESA was conducted on DEGs between MCPIP1 ^fl/fl^ and MCPIP1 ^mko^ BMDMs in the ferroptosis signaling pathway. The GESA plot D) and grouped clustering heatmap E) presented the results. F) Genes with significant differential expression in the ferroptosis signaling pathway are depicted, with red (increased), blue (decreased), and green (no significant difference).

To elucidate the intrinsic regulatory mechanisms of MCPIP1 in macrophages, we performed RNA sequencing on BMDMs from MCPIP1 ^fl/fl^ and MCPIP1 ^mko^ mice. The volcano plot and heatmap (**Figure** **7**A,B) revealed significant differences in gene expression between the two groups. While polarization‐related gene changes may be subtle due to the lack of stimulation, this analysis still offers valuable insights into the potential effects of MCPIP1 deficiency on macrophage polarization in an unstimulated state (Figure 7B). KEGG analysis showed significant enrichment of the ferroptosis pathway among DEGs (Figure 7C). Gene Set Enrichment Analysis (GSEA) indicated that ferroptosis‐related genes were activated in MCPIP1‐deficient macrophages (Figure 7D,E). Specifically, the Xc^−^ system genes (SLC3A2 and SLC7A11) were downregulated in MCPIP1 ^mko^ macrophages compared to controls (Figure 7F).

**Figure 8:**
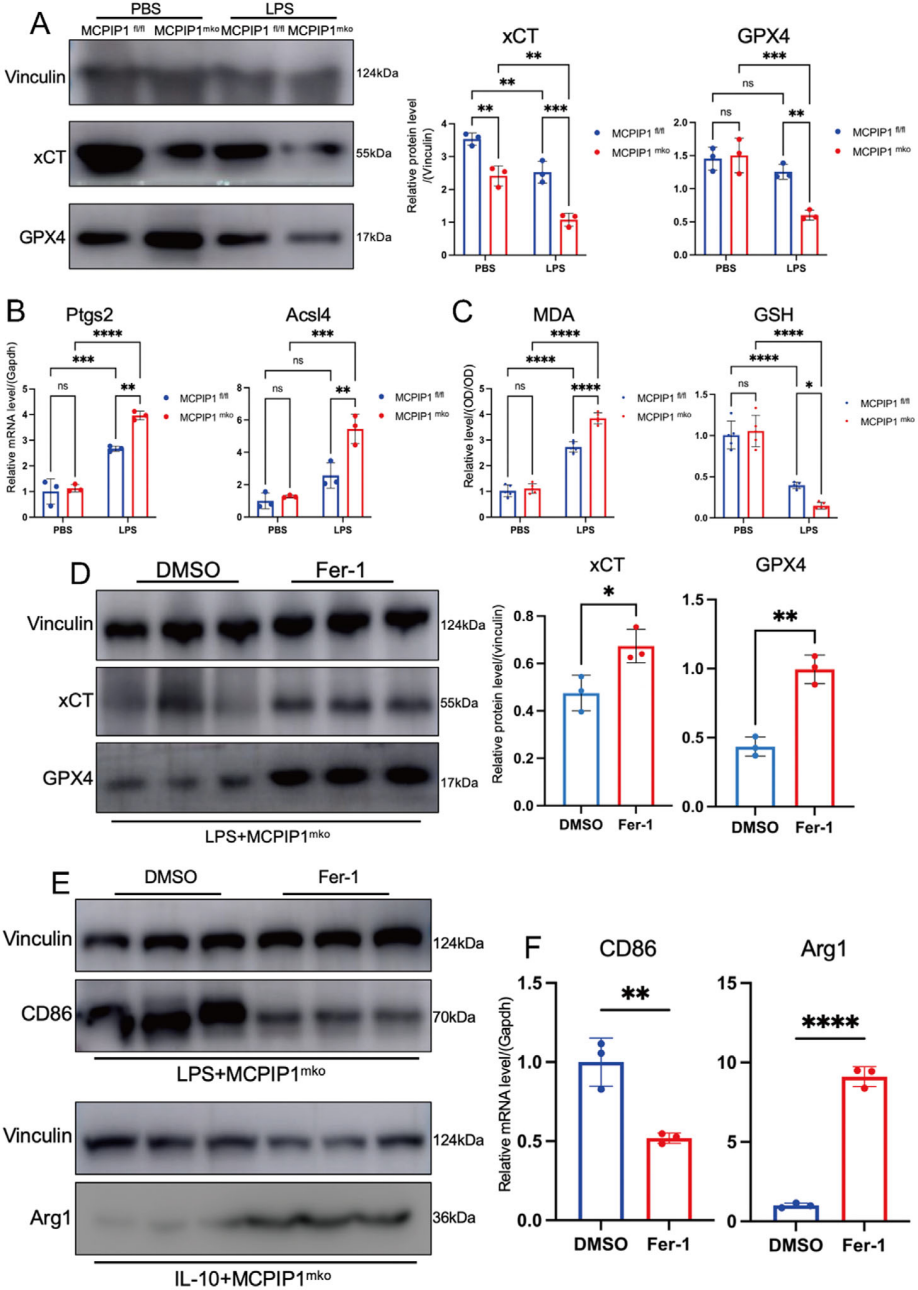
Fer‐1 Attenuates Ferroptosis in MCPIP1‐Deficient Macrophages. A) WB showed protein levels of xCT and GPX4 in MCPIP1 ^fl/fl^ and MCPIP1 ^mko^ BMDMs treated with PBS and LPS. *n* = 3. B) qRT‐PCR measured mRNA levels of Ptgs2 and Acsl4. *n* = 3. C) Spectrophotometric analysis determined relative levels of MDA and GSH. *n* = 5. D) Protein levels of xCT and GPX4 in MCPIP1 ^mko^ BMDMs after LPS (50 ng mL^−1^,24 h), with Fer‐1 (2µm,6 h) treatment. *n* = 3. E) Protein levels and mRNA levels of M1 and M2 macrophage markers, CD86 and Arg1, respectively, in BMDMs after Fer‐1 treatment. *n* = 3. ns *P* > 0.05, ^*^ *P* < 0.05, ^**^ *P* < 0.01, ^***^ *P* < 0.001, ^****^ *P* < 0.0001.

Further experiments showed reduced xCT (SLC7A11) and GPX4 levels in MCPIP1 ^mko^ macrophages, especially under the LPS treatment (**Figure** **8**A). Additionally, mRNA levels of Ptgs2 and Acsl4, which promote ferroptosis, were significantly increased in MCPIP1 ^mko^ BMDMs (Figure 8B). Malondialdehyde (MDA) and glutathione (GSH) levels did not differ between untreated groups but showed significant differences after LPS treatment (Figure 8C). Treatment with Fer‐1, a classic ferroptosis inhibitor, restored xCT and GPX4 levels (Figure 8D) and decreased the M1 marker (CD86) (Figure 8E). Furthermore, MCPIP1‐deficient macrophages, which initially struggled to differentiate into the M2 phenotype, successfully underwent this transition after Fer‐1 treatment (Figure 8E). These findings indicated that ferroptosis serves as a critical signaling pathway through which MCPIP1 regulates phenotypic transformation of macrophages.

**Figure 9:**
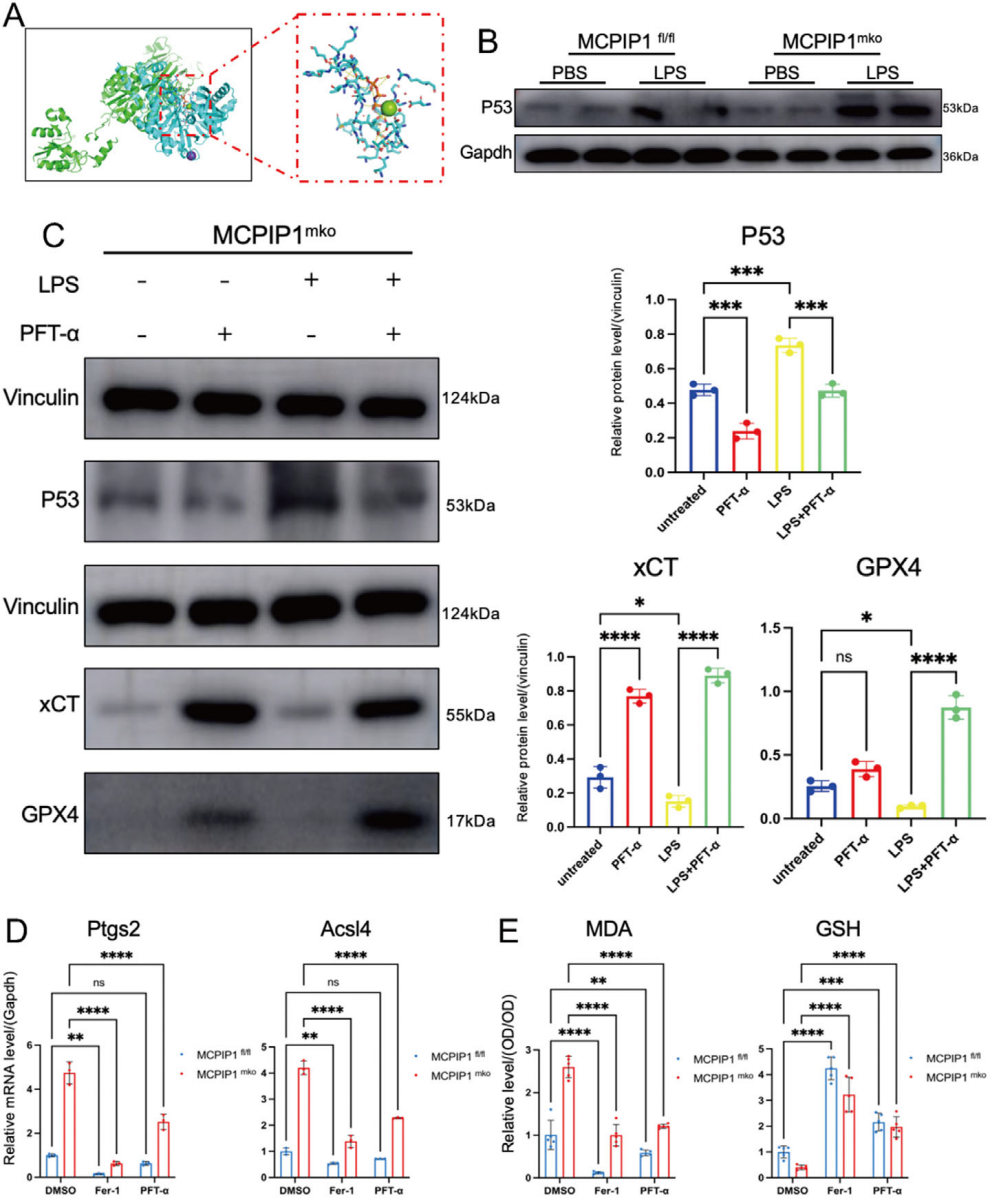
MCPIP1 Regulates Ferroptosis Through P53. A) Z‐dock prediction of the docking model between MCPIP1 and P53, with MCPIP1 in green, P53 in blue, and the binding domain highlighted in a red box. B) WB showed P53 in MCPIP1 ^fl/fl^ and MCPIP1 ^mko^ BMDMs after treatment with PBS and LPS. *n* = 3. C) WB analysis of protein levels of xCT and GPX4 in MCPIP1 ^mko^ BMDMs after LPS (50 ng mL^−1^, 24 h) and PFT‐α (10µm,6 h) treatment. *n* = 3. D) qRT‐PCR measured mRNA levels of Ptgs2 and Acsl4 in MCPIP1 ^fl/fl^ and MCPIP1 ^mko^ BMDMs under DMSO, Fer‐1 and PFT‐α treatment. *n* = 3. E) Spectrophotometric analysis was employed to determine the relative levels of MDA and GSH in MCPIP1 ^fl/fl^ and MCPIP1 ^mko^ BMDMs treated with DMSO, Fer‐1 and PFT‐α. *n* = 5. ns *P* > 0.05, ^*^ *P* < 0.05, ^**^ *P* < 0.01, ^***^ *P* < 0.001, ^****^ *P* < 0.0001.

**Figure 10:**
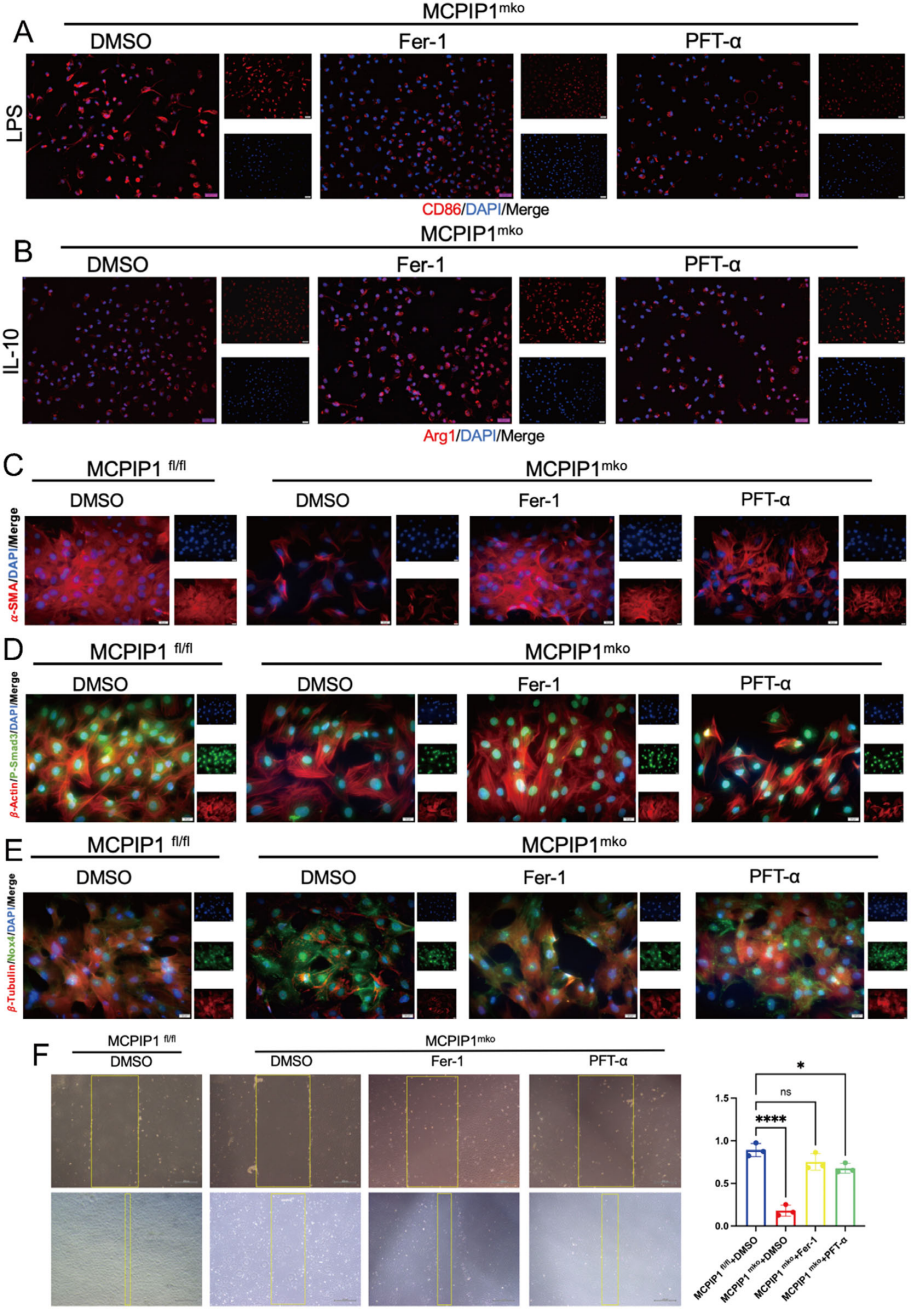
Inhibition of Ferroptosis in Macrophages Promotes Their Maturation and Activates Cardiac Fibroblasts. A) MCPIP1^mko^ BMDMs were pretreated with LPS and then treated with DMSO, Fer‐1, or PFT‐α. The expression level of CD86 was detected by IF. DAPI (blue), CD86 (red). B) MCPIP1 ^mko^ BMDMs were pretreated with IL‐10 and then treated with DMSO, Fer‐1, or PFT‐α. The expression level of Arg1 was detected by IF. DAPI (blue), Arg1 (red). C) The expression level of α‐SMA in PCFs cocultured with BMDMs was detected by IF. DAPI (blue), α‐SMA (red). D) Similarly, P‐Smad3 in PCFs was detected. DAPI (blue), β‐actin (red), P‐Smad3 (green). E) Again, NOX4 in cardiac fibroblasts was detected. DAPI (blue), β‐Tubulin (red), NOX4 (green). F) A wound‐healing assay was performed to assess the proliferation and migration capabilities of PCFs. *n* = 3.ns *P* > 0.05, ^*^ *P* < 0.05, ^**^ *P* < 0.01, ^***^ *P* < 0.001, ^****^ *P* < 0.0001.

Given that MCPIP1 significantly inhibits the Xc^−^ system in macrophages, P53 emerged as a potential target for MCPIP1 regulation (Figure 7F). The protein‐binding prediction model Z‐dock was used to predict the binding sites between MCPIP1 and P53.^[^ ^30^ ^]^ The model suggested extensive interactions between MCPIP1 and P53, facilitating the formation of a stable complex (**Figure** **9**A). Consistent with this, WB results showed that MCPIP1 deficiency elevated P53 protein levels in macrophages (Figure 9B). Treatment with PFT‐α, a P53 inhibitor,^[^ ^31^ ^]^ restored xCT and GPX4 levels (Figure 9C). Both Fer‐1 and PFT‐α significantly decreased mRNA levels of Ptgs2, Acsl4 (Figure 9D) and MDA levels while increasing GSH levels in BMDMs (Figure 9E). IF showed that both Fer‐1 and PFT‐α reduced M1 marker (CD86) expression and promoted M2 marker (Arg1) expression (**Figure** **10**A,B). However, Fer‐1 appeared to be more effective than PFT‐α.

The effects of inhibiting ferroptosis in MCPIP1‐deficient macrophages on fibroblast activation was also examined. IF results after co‐culture indicated that MCPIP1 ^mko^ BMDMs inhibited the activation of PCFs into myofibroblasts, as evidenced by decreased α‐SMA expression (Figure 10C). The TGF‐β/Smad3 signaling pathway within fibroblasts was suppressed, as shown by reduced nuclear translocation of P‐Smad3 (Figure 10D), which is detrimental to collagen I (Col1) secretion. Oxidative stress within PCFs increased, as indicated by elevated NOX4 expression (Figure 10E), and their migration and proliferation capabilities declined (Figure 10F). Treatment with Fer‐1 or PFT‐α reversed these adverse effects on PCFs (Figure 10C–F).

These findings highlight the role of MCPIP1 in inhibiting ferroptosis through the regulation of P53 and the Xc^−^ system, thereby maintaining ferroptosis‐related gene expression and balancing macrophage polarization.

### 2.6 Splenectomy Alleviates Cardiac Damage Caused by MCPIP1 Deficiency

**Figure 11:**
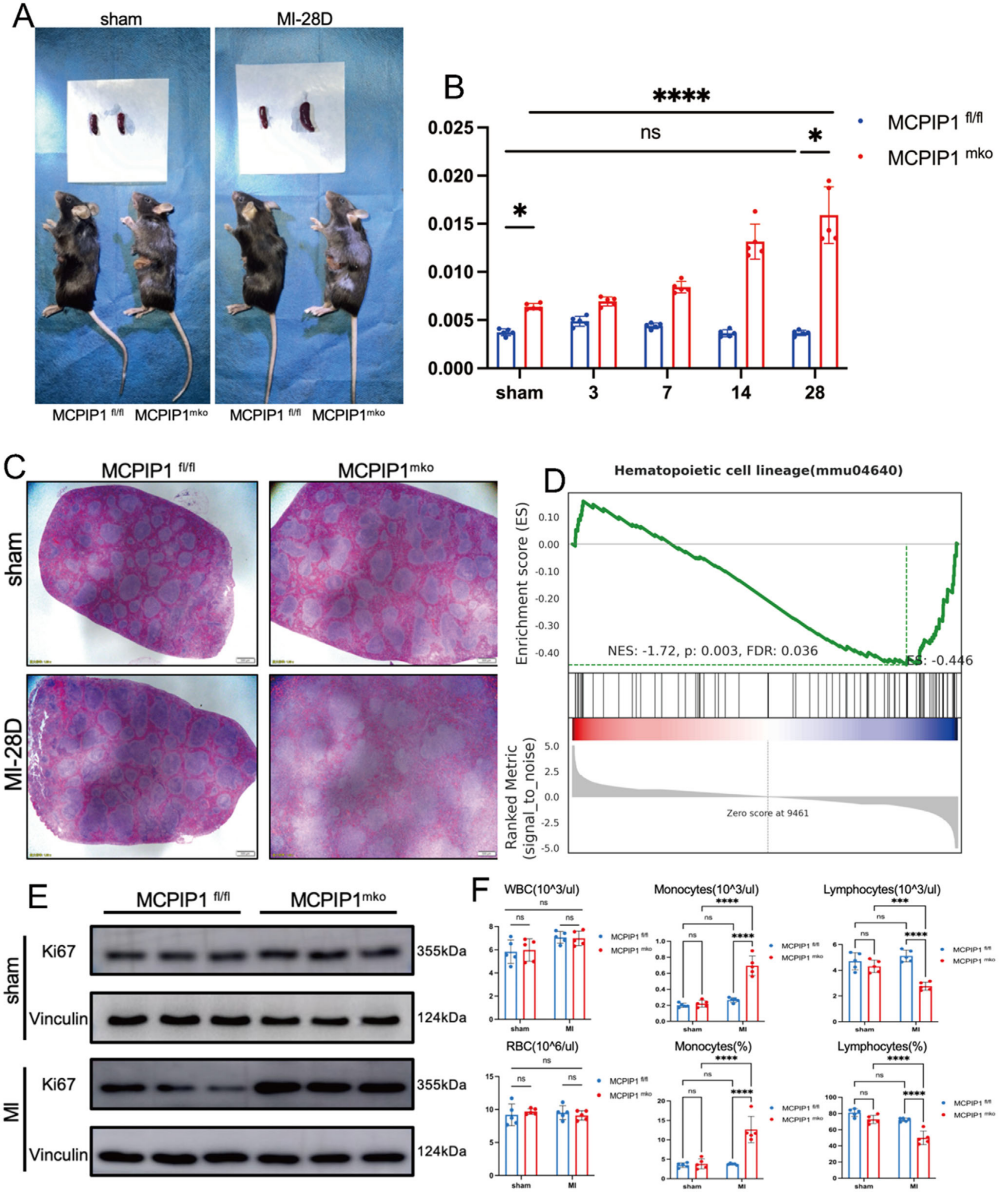
Hyperactive Hematopoietic Function in MCPIP1 ^Mko^ Mice Spleen. A) Necropsy showed spleen size alterations post‐MI. B) Spleen‐to‐body weight ratio at different time points post‐MI (sham, 3, 7, 14, 28 days). C) HE staining revealed histological changes in splenic architecture post‐MI. D) GSEA enrichment analysis of hematopoietic cell lineage‐associated genes using DEGs in BMDMs from MCPIP1 ^fl/fl^ and MCPIP1 ^mko^ mice. E) WB analysis of Ki67 protein levels in the spleen post‐MI. F) Complete blood cell counts in MCPIP1 ^fl/fl^ and MCPIP1 ^mko^ mice pre‐ and post‐MI. ns *P* > 0.05, ^*^ *P* < 0.05, ^**^ *P* < 0.01, ^***^ *P* < 0.001, ^****^ *P* < 0.0001.

The significant splenomegaly observed in MCPIP1 ^mko^ mice following MI is a critical phenomenon. Gross dissection revealed marked splenic enlargement in MCPIP1 ^mko^ mice (**Figure** **11**A). In control mice, there was no significant difference in spleen‐to‐body weight ratio between the sham group (0.003740 ± 0.000356 g g^−1^) and the MI‐28D group (0.003677 ± 0.000280 g g^−1^) (Figure 11A). However, MCPIP1 ^mko^ mice showed a substantial increase from the sham group (0.006394 ± 0.000326 g g^−1^) to the MI‐28D group (0.015898 ± 0.002947 g g^−1^, *P* < 0.0001) (Figure 11B). H&E staining showed significant expansion of the red pulp structure and disrupted splenic architecture in MCPIP1 ^mko^ mice, indicating hypersplenism (Figure 11C). RNA‐seq revealed significant activation of hematopoietic cell lineage genes and signaling pathways in MCPIP1‐deficient BMDMs (Figure 11D). To validate whether splenic hematopoietic activity was activated, we measured the levels of Ki‐67, a critical marker for hematopoietic stem cell activation in the spleen (Figure 11E). WB results demonstrated a marked increase in Ki‐67 expression in the spleens of MCPIP1 ^mko^ mice following MI. Blood analysis revealed that MCPIP1 ^mko^ mice had higher absolute and percentage counts of circulating monocytes post‐MI‐28D, accompanied by a decrease in lymphocytes. There were no significant changes in total white blood cell (WBC) or red blood cell (RBC) counts (Figure 11F). These findings suggest a shift in hematopoietic lineage towards monocytes in MCPIP1 ^mko^ mice.

**Figure 12:**
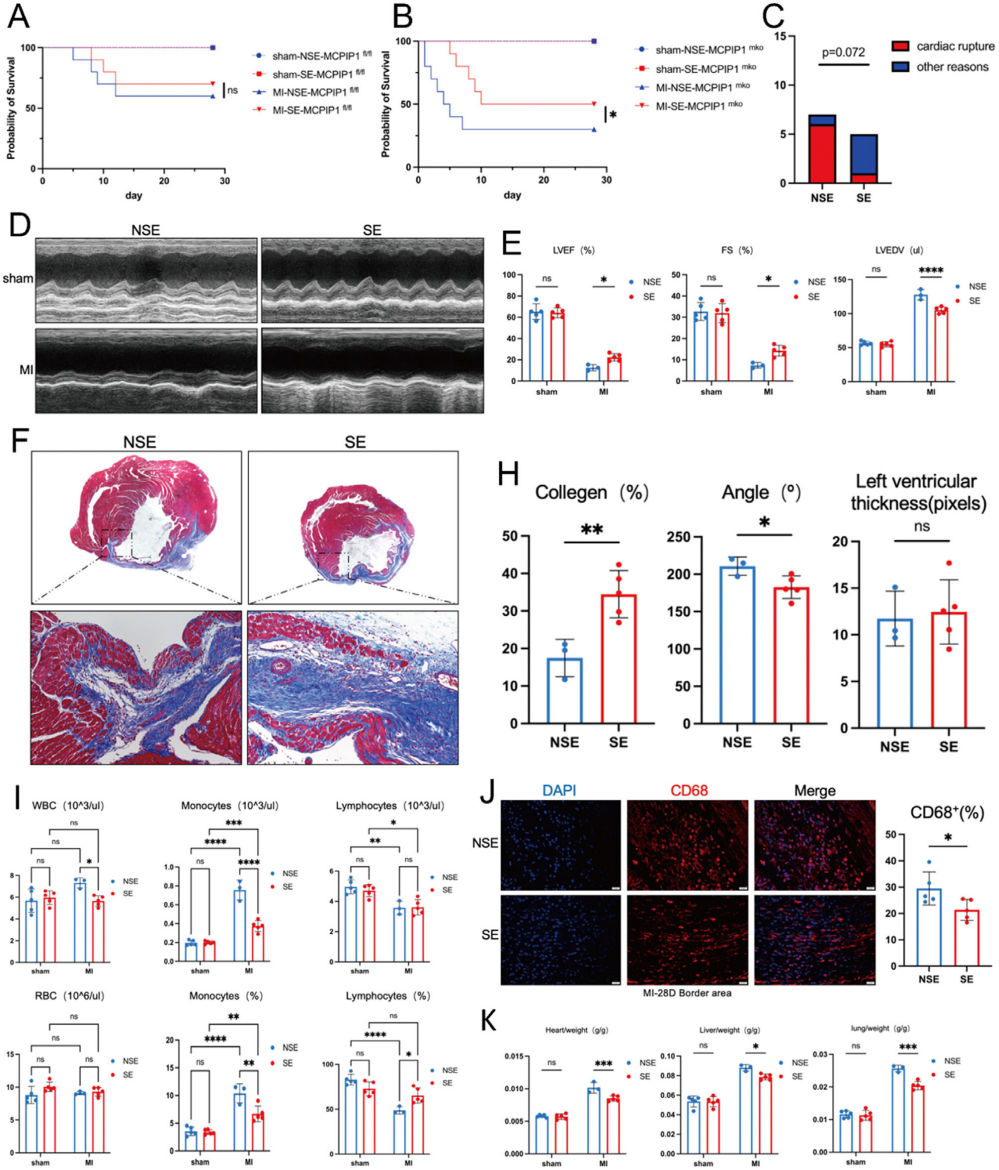
Splenectomy Exerts Protective Effects on Myocardial Infarction in MCPIP1 ^mko^ Mice. A) Survival curve analysis of MCPIP1 ^fl/fl^ mice. B) Survival curve analysis of MCPIP1 ^mko^ mice. C) Effects of splenectomy on the incidence of cardiac rupture in MCPIP1 ^mko^ mice. D) Echocardiographic images of MCPIP1 ^mko^ mice hearts 28 days post‐splenectomy combined with MI. E) The impact of splenectomy on cardiac function in MCPIP1 ^mko^ mice. Each group consists of at least 3 mice. F) Masson's trichrome staining of hearts from MCPIP1 ^mko^ mice 28 days post‐splenectomy combined with MI, to detect collagen content, infarction angle, and left ventricular thickness H). *n* = 5. I) Complete blood cell counts in MCPIP1 ^mko^ mice NSE and SE. J) The percentage of CD68+ cells/ total cell number (counted by nuclei) was calculated. DAPI (blue), CD68 (red), *n* = 5 per field of view. K) The influence of splenectomy on heart‐to‐body weight ratio, liver‐to‐body weight ratio, and lung‐to‐body weight ratio in MCPIP1 ^mko^ mice. Each group comprises at least 3 mice. ns *P* > 0.05, ^*^ *P* < 0.05, ^**^ *P* < 0.01, ^***^ *P* < 0.001, ^****^ *P* < 0.0001.

To investigate whether the spleen mediates post‐myocardial infarction (MI) inflammatory regulation and monocyte proliferation, splenectomy was performed concomitantly with MI modeling. In MCPIP1 ^fl/fl^ mice, splenectomy (SE) did not significantly affect survival rates compared to non‐splenectomized (NSE) controls (**Figure** **12**A). However, in MCPIP1 ^mko^ mice with MI, splenectomy significantly improved survival rates, delaying the time to death in the acute phase (5 days post‐MI) compared to NSE mice (Figure 12B). Although the rate of cardiac rupture was higher in the NSE group (85%) than in the SE group (20%), this difference was not statistically significant (P = 0.072) (Figure 12C). Regarding cardiac function 28 days post‐MI, SE MCPIP1 ^mko^ mice showed improvements in LVEF, FS, and LVEDV compared to NSE mice (Figure 12D,E). As for cardiac structure, HE revealed that splenectomy increased cardiac collagen content and reduced infarct size in MCPIP1 ^mko^ mice, whereas no significant difference was observed in left ventricular wall thickness (Figure 12F,H). The circulating monocytosis induced by MI in MCPIP1 ^mko^ mice was restored following splenectomy (Figure 12I). In addition to circulating monocytes, macrophages in the IBZ were also markedly reduced (Figure 12J). Due to the significant improvement in cardiac pumping function after splenectomy, the blood stasis in the heart, lungs, and liver of MCPIP1 ^mko^ mice also improved (Figure 12K).

## 3 Discussion

**Figure 13:**
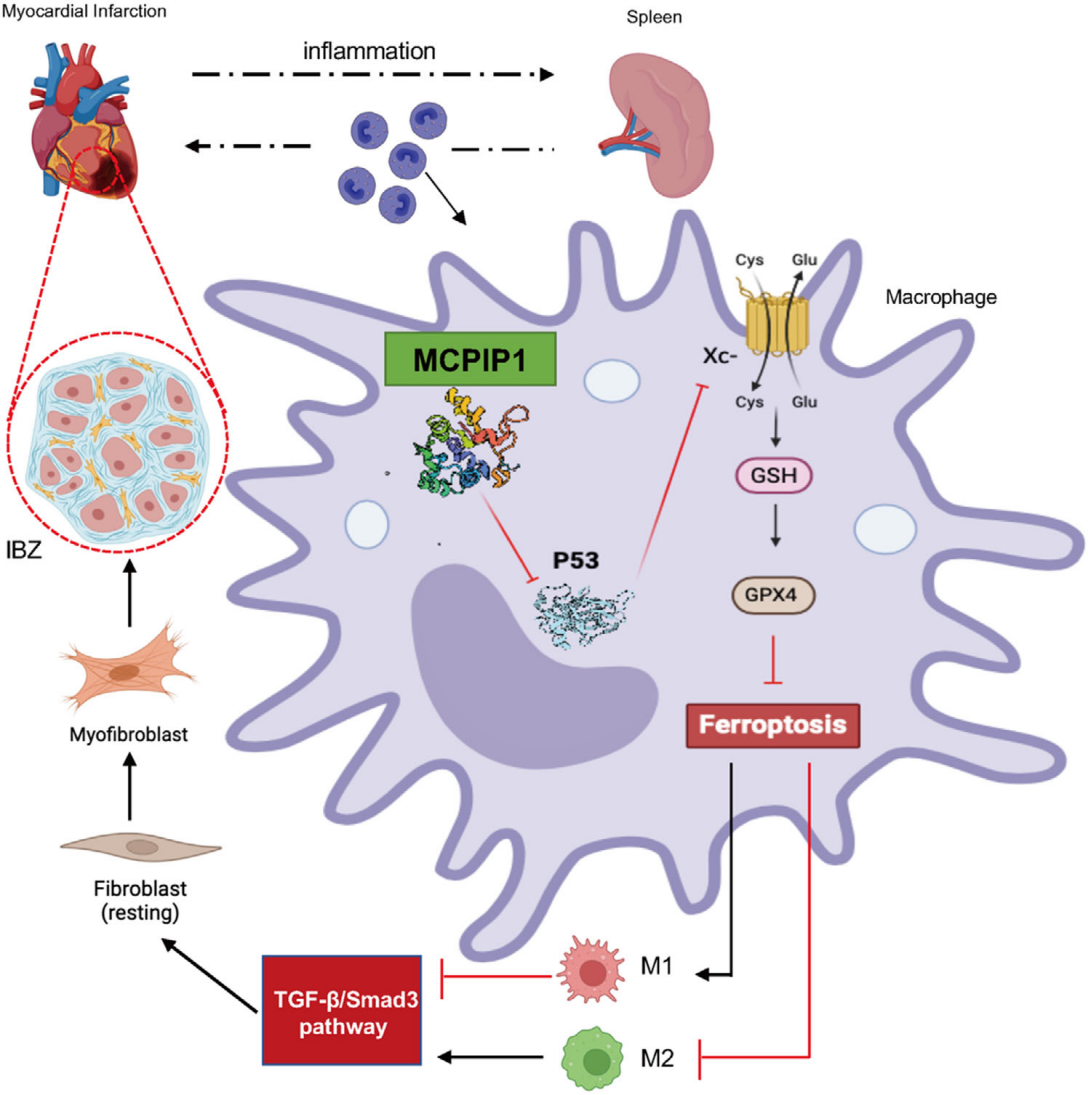
Diagram of the Regulatory Mechanism of MCPIP1. Following the occurrence of MI, necrotic cardiomyocytes release inflammatory cytokines into the bloodstream, leading to the recruitment of macrophages. This intense inflammatory response, albeit short‐lived, not only stimulates hematopoiesis in the bone marrow but also in the spleen. Due to the deficiency of MCPIP1 in macrophages, a substantial number of macrophages recruited to the IBZ undergo ferroptosis, which potentiates the M1 phenotype while inhibiting the M2 phenotype. Persistent cardiac inflammation results in the expansion of the red pulp in the spleen and a continuous release of monocytes/macrophages into the circulation. Furthermore, the abundance of inflammatory mediators and the scarcity of TGF‐β in the IBZ hinder the activation of fibroblasts, rendering the repair of the ECM inefficient and slow. Collectively, these factors contribute to a severe decline in cardiac function, a marked increase in the rate of heart rupture, and an extremely low survival rate in MCPIP1 ^mko^ mice. Diagram was created in https://BioRender.com.

After myocardial infarction, a controlled inflammatory response is essential for clearing necrotic tissue and cellular debris from the infarcted area.^[^ ^32^ ^]^ This response requires the coordinated action of multiple cell types and organs.^[^ ^33^ ^]^ Our research demonstrates that MCPIP1 plays a central role in regulating this process by modulating macrophage polarization, fibroblast activation, and splenic immune cell output. This multifaceted regulation highlights MCPIP1's critical function in balancing inflammation and repair after MI (**Figure** **13**).

### 3.1 MCPIP1 Facilitates Macrophage Transition from M1 to M2 Phenotype by Inhibiting P53/Ferroptosis

Ferroptosis is an iron‐dependent regulated cell death mechanism driven by lipid peroxide accumulation and redox imbalance, implicated in cardiovascular pathologies such as atherosclerosis, myocardial infarction, and cardiomyopathy. Our study reveals that MCPIP1 disrupts the pathogenic M1 macrophage‐ferroptosis cycle by degrading P53 to restore Xc⁻ system functionality and suppress ferroptosis. MCPIP1‐deficient macrophages exhibit elevated Ptgs2/Acsl4 mRNA levels and ROS accumulation, sustaining a self‐amplifying loop of lipid peroxidation and pro‐inflammatory cytokine production. Inhibition of ferroptosis (Fer‐1 or PFT‐α) restored Arg1 expression and enabled M2 polarization in MCPIP1‐deficient macrophages, confirming ferroptosis suppression as essential for phenotype switching.

Mechanistically, DAMPs activate macrophage P53 expression, which suppresses the Xc⁻ system to deplete GSH and induce ferroptosis. MCPIP1 counteracts this process through P53 degradation, rebalancing redox homeostasis. While MCPIP1‐deficient macrophages readily adopt LPS‐induced M1 polarization, they resist IL‐10‐mediated M2 conversion. Crucially, P53/ferroptosis inhibition restores M2 transition capacity in these cells, a prerequisite for inflammation resolution and post‐MI tissue repair. These findings position MCPIP1 as a dual regulator of macrophage polarization and ferroptosis, offering therapeutic potential for modulating the inflammation‐ferroptosis axis in cardiovascular recovery.

### 3.2 MCPIP1 Regulates Fibroblast Activation Through the TGF‐β/Smad3 Pathway

Macrophage‐cardiac fibroblast crosstalk critically influences post‐infarction cardiac remodeling. Impaired α‐SMA and collagen I secretion by fibroblasts compromise IBZ structural integrity, predisposing to left ventricular rupture. Prolonged inflammatory responses suppress fibroblast activation, enhance ECM degradation, and reduce collagen deposition, collectively weakening myocardial tensile strength. MCPIP1 deficiency exacerbates this pathology through diminished TGF‐β secretion from macrophages, impairing Smad3‐mediated fibroblast activation and collagen production. Masson's staining demonstrated significantly reduced collagen deposition in MCPIP1‐deficient mice hearts, correlating with elevated cardiac rupture incidence. MCPIP1 ^mko^ BMDMs fail to activate the TGF‐β/Smad3 pathway in fibroblasts, evidenced by reduced α‐SMA expression and suppressed P‐Smad3 nuclear translocation. Co‐culture experiments revealed dual defects: MCPIP1‐deficient BMDMs not only insufficiently stimulated cardiac fibroblasts but also upregulated fibroblast apoptosis genes. Ferroptosis inhibition (Fer‐1/PFT‐α) reversed these effects by restoring TGF‐β signaling, enhancing collagen synthesis, and reducing oxidative stress markers like NOX4.

MCPIP1‐deficient macrophage accumulation in the IBZ, creating a vicious cycle of insufficient fibroblast activation, impaired ECM remodeling, and increased apoptosis susceptibility. Our data establish MCPIP1 as a master regulator bridging macrophage polarization and fibroblast functionality through TGF‐β/Smad3 activation. Therapeutic targeting of this axis may prevent cardiac rupture by promoting myofibroblast differentiation and collagen matrix stabilization.

### 3.3 MCPIP1 Regulates the Cardiosplenic Axis by Controlling Splenic Macrophage Output

The spleen orchestrates systemic cardiac inflammation through the cardiosplenic axis, mediating crosstalk via immune cell trafficking, neural signaling, and cytokine release. Beyond its hematopoietic functions, the spleen serves as a key reservoir for extramedullary monocyte/macrophage production, particularly within the red pulp. Post‐MI, sympathetic activation mobilizes splenic monocytes/macrophages, which infiltrate the IBZ. Our findings identify MCPIP1 as a central regulator of cardiosplenic axis, curbing splenic hyperactivity. MCPIP1‐deficient mice developed hypersplenism, exhibiting 4‐fold spleen weight increases 28 days post‐MI, with red pulp expansion and reduced white pulp areas. This correlated with systemic monocytosis, lymphocytopenia, and chemokine pathway activation via GSEA analysis. Splenic overactivity in MCPIP1 ^mko^ mice drove continuous monocyte/macrophage release into circulation, with these cells preferentially adopting pro‐inflammatory M1 polarization in the IBZ. Splenectomy reversed this pathology, reducing infarct size, improving survival, and preserving cardiac function—particularly in MCPIP1 ^mko^ mice. The intervention attenuated inflammatory macrophage influx, mitigating border zone inflammation critical for determining final infarct dimensions.

MCPIP1 loss disrupts homeostatic control of splenic hematopoiesis, enabling unchecked monocyte production and chemotaxis toward infarct‐associated chemokines. Persistent M1 polarization of splenic‐derived macrophages exacerbates myocardial damage by sustaining inflammatory cascades. These results highlight the spleen's dual role as both a source of reparative cells and a driver of maladaptive inflammation post‐MI, contingent on MCPIP1‐mediated regulation. Clinically, these findings suggest two translational avenues: 1) Assessing MCPIP1 expression in circulating macrophages as a biomarker of cardiosplenic axis dysregulation post‐MI, and 2) Developing therapies targeting splenic macrophage output to modulate infarct zone inflammation. Strategic modulation of this axis could complement existing interventions by balancing immune‐driven repair and destructive inflammation during cardiac recovery.

## 4 Conclusion 

Our study has uncovered several pivotal insights that position MCPIP1 as a crucial regulator in the inflammatory and reparative processes following myocardial infarction. Firstly, MCPIP1 enables the transition of macrophages from the pro‐inflammatory M1 phenotype to the reparative M2 phenotype by inhibiting the P53/ferroptosis signaling pathway. Secondly, within the IBZ microenvironment, MCPIP1 enhances the secretion of TGF‐β by macrophages, which in turn activates fibroblasts into myofibroblasts. Lastly, MCPIP1 plays a critical role in regulating splenic monocyte output following MI. Collectively, these findings underscore MCPIP1 as a promising therapeutic target for future strategies aimed at mitigating inflammation, promoting tissue repair, and improving cardiac function following MI. Targeting MCPIP1 could provide a novel approach to modulate macrophage polarization, fibroblast activation, and splenic immune cell output, thereby enhancing the overall recovery and prognosis in patients with myocardial infarction.

## 5 Limitations and Future Directions

A significant limitation of this study is the lack of a targeted delivery system for ferroptosis inhibitors and p53 inhibitors specifically to macrophages in mice. This gap precluded the inclusion of relevant animal experiments that could have further validated our findings. The development of effective macrophage‐specific delivery strategies is therefore a crucial direction for future research.

Several potential approaches could address this challenge. For instance, nanoparticle‐based delivery systems, antibody‐drug conjugates, and gene‐editing technologies all hold promise as innovative solutions to enhance precision and therapeutic translation. Advancing these targeted delivery systems will not only provide more comprehensive validation of our findings but also lay the groundwork for clinically viable interventions targeting macrophage polarization and ferroptosis in the context of myocardial infarction.

## 6 Experimental Section

### Data Source and Processing

Gene Expression Omnibus (GEO) database provides datasets including GSE166780 (comparing peripheral blood monocytes from acute myocardial infarction [AMI] patients versus healthy individuals), GSE172270 (comparing monocyte‐derived macrophages from AMI patients versus healthy individuals), and GSE268352 (comparing lipopolysaccharide [LPS]‐treated versus phosphate‐buffered saline [PBS]‐treated human monocyte‐derived macrophages). Differentially expressed genes across these datasets were analyzed using R software (version 4.4.2). Further Gene Ontology (GO) and Kyoto Encyclopedia of Genes and Genomes (KEGG) analyses were conducted on these DEGs. GSE88924 dataset from GEO was also analyzed using Short Time‐series Expression Miner (STEM) analysis to investigate the trend of gene expression of cardiac macrophages, following the time sequence of MI1D‐MI7D‐MI14D. Genes lacking significant temporal expression gradients were excluded, and the remains were categorized into different modules. False Discovery Rate with adjusted p‐value <0.05 was considered statistically significant.

### Mice

The C57BL/6 MCPIP1^fl/fl^ and C57BL/6 LysM‐Cre mice have been previously characterized. These mice were reared under pathogen‐free conditions at the animal facilities of Tongji Medical College. Animal experiments were conducted according to the Chinese National Institutes of Health and Animal Care guidelines and were approved by the Animal Care Ethics Committee of Shanghai Tenth People's Hospital of Tongji University School of Medicine (approval number: SHDSYY‐2022‐4303). LysM‐Cre mice were crossed with MCPIP1^fl/fl^ mice to generate myeloid‐specific MCPIP1 knockout mice, with co‐housed MCPIP1^fl/fl^ mice serving as controls.^[^ ^34^ ^]^ For simplicity, these knockout mice (MCPIP1^fl/fl^‐LysM‐Cre+) are referred to as MCPIP1 ^mko^ mice hereafter. The following primer sequences were used for genotyping via tail biopsy: floxp (forward primer: CAGGTATGTTGCAAATGGCCCGAC; reverse primer: CTGTAGGACTCAGCCCATGGCC) and LysM‐Cre (forward primer: CCCAGAAATGCCAGATTACG; reverseprimer: CTTGGGCTGCCAGAATTTC‐TC).

### MI Model

The MI model was established following a previously described protocol.^[^ ^35^ ^]^ Briefly, weight‐matched male mice (6 weeks old) were anesthetized and intubated. A skin incision was made on the left side between the third and fourth intercostal spaces, and the muscle and pericardium were separated to expose the left anterior descending (LAD) artery. The LAD was ligated using a 7‐0 silk suture, and the chest was then sutured. Sham‐operated mice underwent the same procedure without LAD ligation. Heart tissues were collected for further experiments.

### Splenectomy

Splenectomy was performed in anesthetized male mice by making a small abdominal incision, isolating the spleen, severing and ligating its vessels and ligaments before the organ removal. Hemostasis was ensured to control bleeding, and postoperative care was provided to monitor for complications and support recovery.

### Echocardiography

Echocardiography evaluated the mice's cardiac function at 10 weeks post‐surgery using the Vevo 2100 system equipped with an 18–30 MHz transducer. Mice were anesthetized with 1%–2% isoflurane, and left ventricle's parasternal long axis and mid‐papillary muscle short axis were recorded for five cardiac cycles. A blinded operator measured left ventricular ejection fraction (LVEF), fractional shortening (FS), and left ventricular end‐diastolic volume (LVEDV).

### Masson Trichrome and H&E Staining

On day 28 post‐MI, heart tissues from 1 mm below the ligation to the apex were paraffin‐embedded. Heart sections were stained with Masson Trichrome and Hematoxylin and Eosin (H&E) to visualize infarcted areas. Images were captured using an Olympus microscope, and infarct size was calculated using ImageJ software.

### BMDMs Preparation

Bone marrow‐derived macrophages (BMDMs) were isolated from the femurs and tibias of female mice (≈6 weeks old) as previously described. These cells were differentiated in DMEM supplemented with 10% fetal bovine serum (FBS) and 20 ng mL^−1^ of MCSF.

### Primary Cardiac Fibroblasts Isolation

Neonatal mouse hearts were dissected, minced, and digested with collagenase II for four 10‐min intervals. The cells were centrifuged and filtered through a 70 µm filter. After plating on 6‐cm dishes for an hour, adherent cells were cultured in DMEM containing 20% FBS in a humidified incubator at 37^ °^C and 5% CO₂.

### RNA‐Seq

RNA was extracted using TRIzol and sequenced on an Illumina HiSeqTM 2500. Differentially expressed genes (DEGs) were analyzed using the limma R package, with |Log2 fold change| >1.5 and p‐value <0.05 as threshold. Volcano plots were generated to visualize DEG distribution, and heatmap were used to display specific DEGs. KEGG pathway enrichment analysis was performed to identify enriched biological pathways.

### Immunofluorescence Staining (IF)

Tissues and cells were fixed and permeabilized with 4% paraformaldehyde and 0.1% Triton X‐100 for 10 min at 4^ °^C. After blocking with 5% BSA for 30 min, samples were incubated with primary antibodies overnight at 4^ °^C, followed by Alexa Fluor 488/594‐conjugated secondary antibodies for 1 h at room temperature. Nuclei were stained with DAPI, and images were captured using fluorescence microscopy. Positive cells and mean fluorescence intensity were quantified in randomly selected fields using ImageJ software (version 2.0.0‐RC).

### RNA Isolation and Quantitative Real‐Time Polymerase Chain Reaction

RNA was extracted from heart tissue homogenates and cells using TRIzol reagent. The reverse transcription was performed using the PrimeScript RT Reagent Kit. Quantitative real‐time PCR (qRT‐PCR) was conducted using Hieff qPCR SYBR Green Master Mix on a LightCycler 480 Real‐time PCR System (Roche), following the manufacturer's instructions. Relative gene expression was calculated by the 2‐ΔΔCt method, normalized to GAPDH expression.

### Western Blot (WB)

Proteins were extracted from cells using RIPA buffer, and concentrations were measured with a BCA protein assay kit. Samples were then resolved by SDS‐PAGE and transferred onto a PVDF membrane (Millipore). After blocking with 5% nonfat milk containing 0.1% Phosphate‐Buffered Saline with Tween‐20 (PBST) for 1 h at room temperature, membranes were incubated with primary antibodies overnight at 4^ °^C. Following PBST washes, membranes were incubated with the appropriate secondary antibody, and signals were detected using ECL Western Blotting Detection Reagent.

### Statistical Analysis

Data are presented as means ± standard deviations. Normality was assessed using the Shapiro‐Wilk test. For two‐groups comparisons, a two‐tailed Student's t‐test was used if data were normally distributed; otherwise, a Mann‐Whitney U test was applied. For three or more groups, a one‐way ANOVA followed by the Bonferroni post hoc test was used for normally distributed data; otherwise, a Kruskal‐Wallis test with the Dunn post hoc test was applied. Survival rate changes were analyzed using the Kaplan–Meier method and log‐rank test. GraphPad Prism 7.0 and SPSS 20.0 were used for all statistical analyses. A two‐sided P value <0.05 (ns *P* > 0.05, ^*^ *P* < 0.05, ^**^ *P* < 0.01, ^***^ *P* < 0.001, ^****^ *P* < 0.0001) indicates statistical significance.

## Conflict of Interest

The authors declare no conflict of interest.

## Data Availability

The data that support the findings of this study are available from the corresponding author upon reasonable request.
